# Adenoma of the Nipple: A Case Report

**DOI:** 10.7759/cureus.64105

**Published:** 2024-07-08

**Authors:** Isabelle S Beaudoin, Abdulrahman Karmach, Ava M Koehler De Celaya, Cyenthia Koehler, Joseph A Di Como

**Affiliations:** 1 Research, St. George's University School of Medicine, Saint George's, GRD; 2 Pathology, Abrazo Arizona Heart Hospital, Phoenix, USA; 3 Breast Surgical Oncology, Ironwood Cancer and Research Centers, Scottsdale, USA

**Keywords:** benign breast mass, breast benign and malignant surgery, benign breast condition, bilateral breast masses, breast histology, paget's disease of the breast, epithelial cell proliferation, invasive ductal breast carcinoma, breast oncology, nipple adenoma

## Abstract

Nipple adenomas are rare, benign breast lesions that present similarly to breast malignancies, often manifesting with unilateral bloody discharge, a palpable mass, and/or nipple distortion. Imaging techniques have limited specificity in distinguishing nipple adenomas from malignancy; therefore, clinicians must rely on histologic and immunohistochemistry evaluation. Here, we highlight the case of a 69-year-old woman with bilateral nipple adenomas presenting as an enlarging nipple mass with chronic nipple discharge. Complete lesion resection with clear margins stands as the primary route of management and complete avoidance of re-occurrence. However, partial excision with nipple preservation has been reported to be successful in selected cases.

## Introduction

Breast cancer represents 32% of new cancer diagnoses in 2024 while accounting for 15% of cancer-related deaths [1]. Nipple adenomas are an uncommon subtype of benign breast tumors, arising from the proliferation of lactiferous duct epithelial cells. Nipple adenomas comprise 0.1-1.7% of benign breast lesions, predominantly affecting women in their 40s and 50s [2]. Particular challenges include differentiating this benign entity from malignant histologic mimics such as tubular carcinoma and low-grade invasive ductal carcinoma among others.

## Case presentation

**Figure 1:**
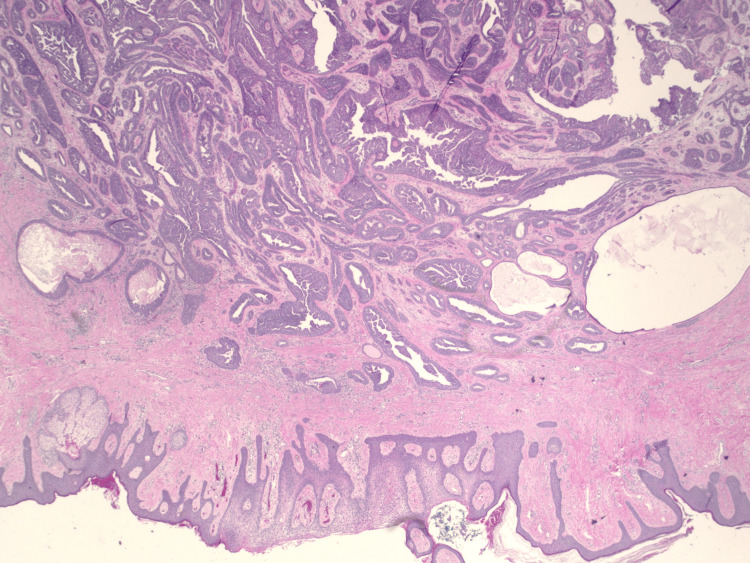
H&E stain of breast nipple ductal adenoma at 20× magnification. H&E: hematoxylin and eosin

**Figure 2:**
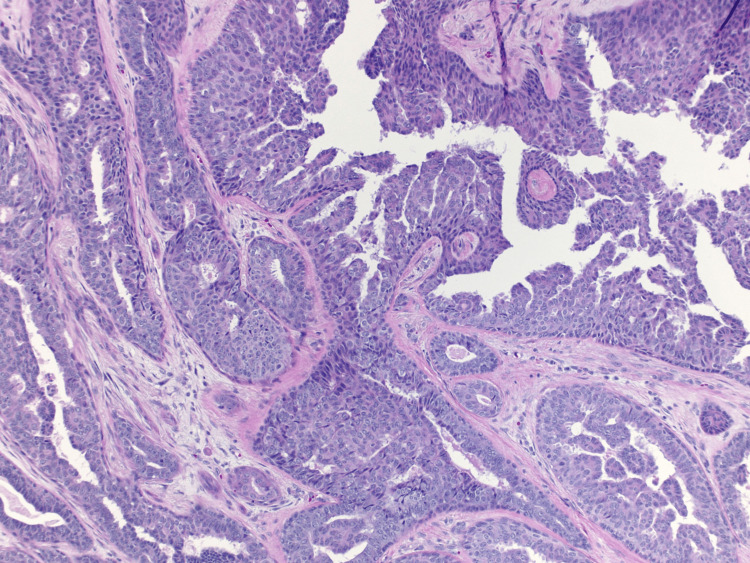
H&E stain of ductal epithelial cell proliferation in breast nipple adenoma at 40× magnification. H&E: hematoxylin and eosin

**Figure 3:**
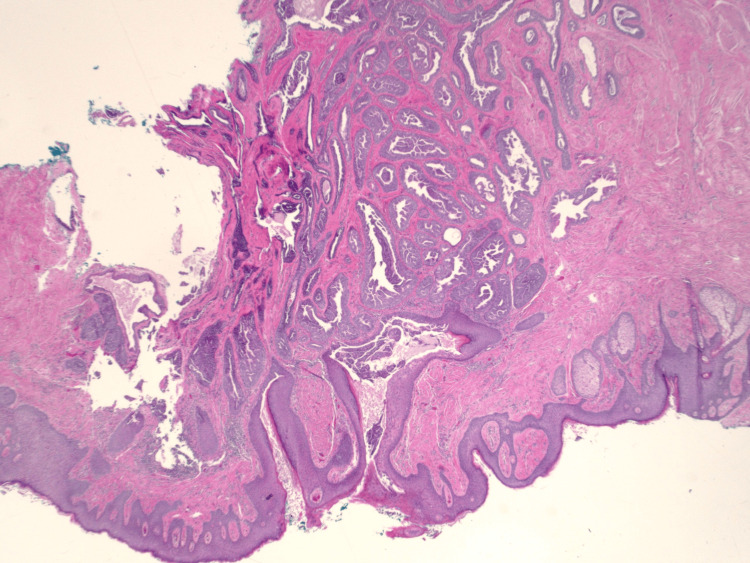
H&E stain of breast nipple ductal adenoma at 20× magnification. H&E: hematoxylin and eosin

**Figure 4:**
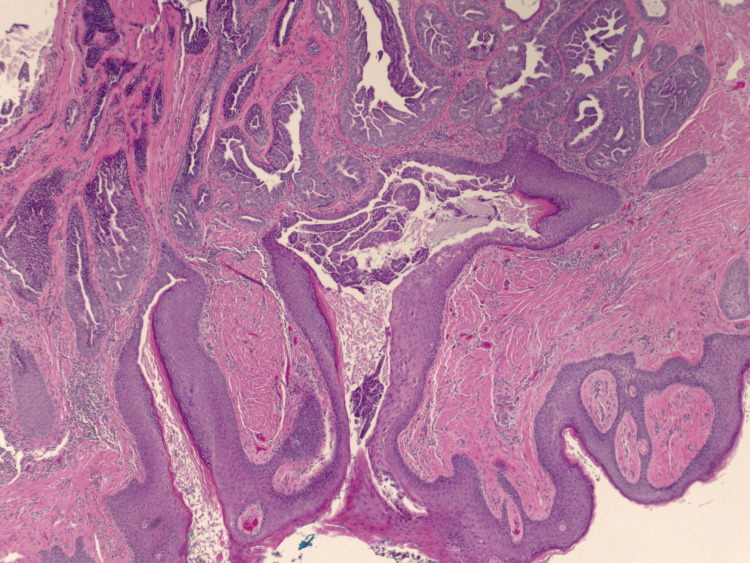
H&E stain of breast duct opening seen in association with breast nipple adenoma at 40× magnification. H&E: hematoxylin and eosin

A 69-year-old female presented to the clinic with complaints of a right nipple mass. The patient reported chronic nipple discharge for several years but recently noticed a mass on the right nipple over the past several weeks. She believed that it had been increasing in size which prompted her to seek medical attention. A diagnostic mammogram with ultrasound demonstrated a 1.1 cm mass within the right breast. A punch biopsy of the nipple was performed, and the results demonstrated a benign adenoma. A bilateral breast MRI exhibited a 12 mm right nipple mass as well as a 7 mm mass within the left nipple. Surgical excision of both masses was recommended and performed. Due to the right adenoma size replacing the entire right nipple, the patient's right nipple was amputated with sparing of the areola. Her final surgical pathology demonstrated bilateral ductal adenomas of the nipple with margins positive for involvement (Figures 1-4). Figure 1 shows the large right breast nipple ductal adenoma with abundant ductal epithelial cell proliferation filling and expanding the ducts. A closer view of this is appreciated in Figure 2 which exhibits the cells growing with a mild mitotic rate that is relatively uniform and benign in appearance. Figure 3 shows the main area of the left breast nipple ductal adenoma, portraying prominent ductal epithelial cell proliferation in a pattern similar to Figure 1 and Figure 2 of the opposite breast. In Figure 4, the breast duct opening associated with the left breast nipple ductal adenoma can be seen. There was no atypia or in situ or invasive disease noted (Figures 1-4). Due to the benign nature of the lesions, the desire to avoid additional surgery, and general cosmetic preferences, the patient ultimately declined additional resection for negative margins and opted for close observation for recurrence.

## Discussion

This case highlights the rare pathologic entity of nipple adenoma. Patients often present with unilateral nipple discharge, crusting, erosion, and/or palpable masses. Additional symptoms include distortion of the nipple's appearance, pain, pruritus, erythema, and ulceration [3]. Nipple discharge is found to be the most common symptom noted by patients, existing in upwards of 58% of cases [2]. Clinical presentation is sometimes indistinguishable from Paget's disease, which can lead to some confusion. Other common differentials include ductal carcinoma in situ, syringomatous adenoma, tubular carcinoma, and invasive papilloma [4]. Awareness of these signs and symptoms is crucial for healthcare providers to consider nipple adenoma in their differential diagnosis. Although benign, there is evidence suggesting a connection with breast carcinoma. In a large review of 224 cases, Abdulwaasey et al. found 16.5% of nipple adenomas to have co-existent breast carcinoma, over half of which arose in the ipsilateral breast [5]. Invasive ductal carcinoma was the most common breast cancer to co-exist with nipple adenoma [5]. Abdulwaasey et al. also reported multiple patients with co-existing nipple adenoma and ductal carcinoma in situ [5]. Standard breast imaging, including ultrasound and mammography, are routinely employed to investigate for malignancy and the extent of the lesion. Mammography can identify a mass but is often unremarkable in the presence of nipple adenoma [6]. Breast ultrasound can yield more specific findings, showing a well-circumscribed mass, described as hypoechoic with internal vascularity [2,7].

However, it is important to note that these imaging modalities are generally not reliable in determining the presence of nipple adenoma because of the similar density of the nipple, adjacent skin, and breast tissue [8]. Histology plays a pivotal role in distinguishing and diagnosing nipple adenomas. Of the various histology patterns, the visualization and preservation of the dual layer of myoepithelial and epithelial cells have been widely accepted to be confirmatory of nipple adenoma, arguing against Paget's disease and invasive ductal carcinoma. Another significant finding is a lack of cellular atypia in the setting of hyperplastic change. Fine needle aspiration typically reveals nuclei uniformity within the epithelial and myoepithelial cell populations [3]. Biopsy of the nipple tissue has therefore been recommended to confirm the diagnosis prior to complete lesion excision [8]. Immunohistochemistry is a diagnostic tool necessary for the confirmation of nipple adenoma. The most common markers include p63, calponin 1, a-smooth muscle actin, h-caldesmon, CK5/6, and CD10 [4]. A positive result of two or more markers has been deemed as diagnostic confirmation, with p63 and CK5/6 antibodies considered as the strongest indicators tied to nipple adenoma. Complete surgical excision has been widely established as the treatment of choice and gold standard of diagnosis for nipple adenoma. Depending on the size of the lesion, surgical excision may entail total resection of the affected nipple. Partial or incomplete excisions of nipple adenomas have been discouraged due to a 25-55% rate of lesion recurrence [3]. Cosmetic satisfaction among patients can pose as an obstacle for treatment. Full disclosure regarding the results of surgical removal, including the post-procedural appearance or lack of nipple, should be clearly communicated to patients. There is little information on alternative, nonsurgical forms of treatment or complications of nipple adenomas left untreated. There is evidence of overtreatment with mastectomy for nipple adenomas, highlighting the importance of biopsy prior to any surgical intervention [9].

## Conclusions

The case of nipple adenoma presented here serves as a clinical presentation for healthcare providers. Recognizing the clinical manifestations, understanding the limitations of imaging, and emphasizing the necessity of histopathologic and immunohistochemical evaluation are critical for accurate diagnosis. Complete surgical excision remains the gold standard for treatment, with careful consideration of aesthetic outcomes. Awareness of the potential association with breast cancer ensures thorough patient evaluation and appropriate management.
